# The aged nonhematopoietic environment impairs natural killer cell maturation and function

**DOI:** 10.1111/acel.12303

**Published:** 2015-02-09

**Authors:** Hesham M Shehata, Kasper Hoebe, Claire A Chougnet

**Affiliations:** Division of Immunobiology, Department of Pediatrics, Cincinnati Children's Hospital Medical Center and the University of CincinnatiCincinnati, OH, 45229, USA

**Keywords:** aging, cellular immunology, cytokines, mouse

## Abstract

Natural killer (NK) cells are critical in eliminating tumors and viral infections, both of which occur at a high incidence in the elderly. Previous studies showed that aged NK cells are less cytotoxic and exhibit impaired maturation compared to young NK cells. We evaluated whether extrinsic or intrinsic factors were responsible for the impaired maturation and function of NK cells in aging and whether impaired maturation correlated with functional hyporesponsiveness. We confirmed that aged mice have a significant decrease in the frequency of mature NK cells in all lymphoid organs. Impaired NK cell maturation in aged mice correlated with a reduced capacity to eliminate allogeneic and B16 tumor targets *in vivo*. This could be explained by impaired degranulation, particularly by mature NK cells of aged mice. Consistent with impaired aged NK cell maturation, expression of T-bet and Eomes, which regulate NK cell functional maturation, was significantly decreased in aged bone marrow (BM) NK cells. Mixed BM chimeras revealed that the nonhematopoietic environment was a key determinant of NK cell maturation and T-bet and Eomes expression. In mixed BM chimeras, NK cells derived from both young or aged BM cells adopted an ‘aged’ phenotype in an aged host, that is, were hyporesponsive to stimuli *in vitro*, while adopting a ‘young’ phenotype following transfer in young hosts. Overall, our data suggest that the aged nonhematopoietic environment is responsible for the impaired maturation and function of NK cells. Defining these nonhematopoietic factors could have important implications for improving NK cell function in the elderly.

## Introduction

It is well established that aging attenuates the host's ability to mount robust immune responses (Dorshkind & Swain, 2009). Immunosenescence is defined as age-dependent alterations that lead to an impaired function in almost every component of the immune system (Frasca & Blomberg, 2009; Haynes & Maue, 2009; Jing *et al*., 2009). A hallmark of immunosenescence is the increased susceptibility of the elderly population to microbial infections (Akbar *et al*., 2004; Maue *et al*., 2009). In addition, the elderly are highly susceptible to the development of tumors with the incidence of cancer being particularly high in individuals aged > 60 years (Campisi, 2003; Jemal *et al*., 2010). Thus, studies geared at providing novel insight into the molecular mechanisms leading to age-related immune dysfunction are critically required to address the need for novel therapeutic interventions for a growing elderly population.

Whereas the function and homeostasis of aged T cells has been extensively investigated, it is becoming increasingly evident that the innate arm of the immune system is also affected in aging. Of particular importance, natural killer (NK) cells play a critical role in coordinating tumor immunosurveillance and the immune response to viral infections (Biron *et al*., 1999).

The function of NK cells is determined by the integration of signals arising from the engagement of their activating and inhibitory receptors (Di Santo, 2006). Such responses are mediated through two major effector NK cell functions, the direct cytolysis of target cells and the production of cytokines and chemokines. NK cell function is also dictated by their stage of maturation, a process that primarily occurs in the bone marrow (BM) (Kumar *et al*., 1979; Seaman *et al*., 1979). During terminal maturation, NK cells become more efficient at eliminating target cells (Kim *et al*., 2002; Fang *et al*., 2008; Chiossone *et al*., 2009) and undergo a process of education (licensing) while acquiring the expression of different Ly49 receptors (Narni-Mancinelli *et al*., 2011). It has been previously reported that NK cell maturation is a 4-stage process that can be described on the basis of surface expression of the TNF superfamily member, CD27 and the integrin, CD11b (Chiossone *et al*., 2009). NK cell maturation starts at the double-negative (DN) stage (CD27^low^ CD11b^low^), which progressively differentiates into immature (CD27^high^ CD11b^low^), then transitional (CD27^high^ CD11b^high^) and finally into mature NK cells (CD27^low^ CD11b^high^), which display a full repertoire of Ly49Rs and the highest cytotoxic potential (Hayakawa & Smyth, 2006; Chiossone *et al*., 2009; Narni-Mancinelli *et al*., 2011).

Previous studies showed that aged human and murine natural killer cells are less cytotoxic *in vitro* and contain reduced proportions of mature NK cells (Nogusa *et al*., 2008, 2012; Fang *et al*., 2010; Almeida-Oliveira *et al*., 2011; Beli *et al*., 2011, 2014; Hazeldine *et al*., 2012; Chiu *et al*., 2013). In addition, it has been reported that the mature subset of aged NK cells have an impaired capacity to home to draining lymph nodes in the context of ectromelia virus (ECTV) infection (Fang *et al*., 2010). However, whether defective NK cell maturation in aging influences their functional competence remains poorly understood. Here, we sought to provide insight into: (i) Whether extrinsic or intrinsic factors are responsible for the impaired maturation and function of NK cells in aging and (ii) whether there is a correlation between the impaired maturation of NK cells and their reduced functional competence in aging. Our novel findings show that the aged nonhematopoietic environment is responsible for the impaired maturation and function of NK cells.

## Results

### NK cells in aged mice are less efficient at eliminating allogeneic and tumor targets *in vivo*

**Figure 1:**
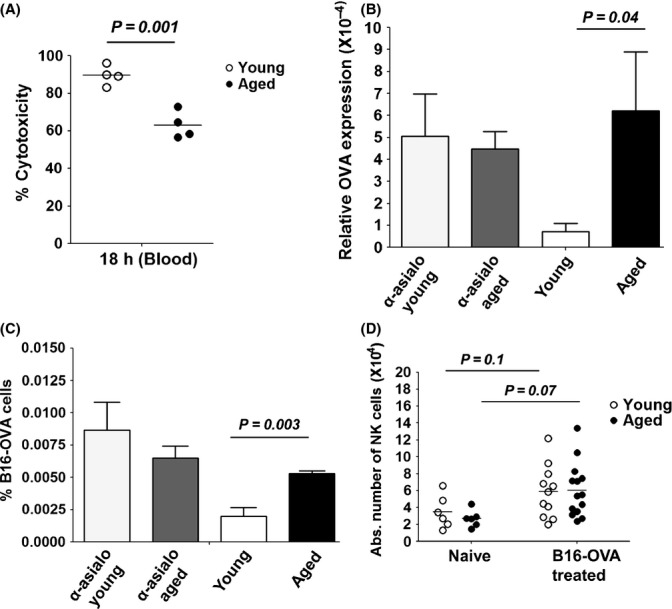
Aged NK cells have a reduced *in vivo* cytotoxic capacity. Aged mice, young mice, and NK cell-depleted mice (anti-asialo GM1 treated) were challenged intravenously with CFSE-labeled allogeneic targets or CFSE-labeled B16 melanoma cells expressing OVA. After 18 h, the efficiency of allogeneic target cell elimination was evaluated by flow cytometry and normalized to anti-asialo-treated recipients. (A) The difference in the mean percentage cytotoxicity between young and aged NK cells. B16-OVA tumor burden in the lungs was evaluated 1 h. postchallenge by (B)*,*RT–PCR for OVA expression and (C)*,* flow cytometry for CFSE expression and compared to NK cell-depleted mice. (D) The absolute number of NK cells (CD3- NKp46+) in the lungs of naïve (*n *=* *6) and B16-OVA challenged young, aged mice (*n *=* *11–14). Data are represented as the mean± SE of two independent experiments with *n *=* *4 mice for young and aged mice and *n *=* *2 for anti-asialo GM1-treated mice. The *P* values (unpaired *t*-tests) represent the difference between aged and young mice. Means are shown as horizontal lines with each point representing one individual mouse. The *P* values between naïve and B16 challenged mice were calculated using paired *t*-tests.

The functional competence of aged NK cells was first determined *in vivo* by testing whether they could eliminate allogeneic (Balb/c splenocytes) targets. Differential CFSE labeling allowed the tracking of allogeneic (Balb/c) versus syngeneic (BL/6) targets in recipient animals. Relative allogeneic cytotoxicity (normalized to NK cell-depleted recipients) was significantly decreased in aged animals (Fig.1A). As expected, syngeneic (BL/6) targets were not eliminated by NK cells in recipient mice (data not shown).

To assess whether this functional impairment also extended to a reduced recognition of tumor cells in aged mice, we next assessed the ability of aged mice to eliminate B16 melanoma cells *in vivo*. B16 melanoma cells, which downregulate the expression of MHC-I (Seliger *et al*., 2001), have been shown to be rapidly eliminated from the lung by NK cells as early as 1 h post-i.v challenge, thus presenting a reliable model to test the functional capacity of aged NK cells (Grundy *et al*., 2007). Accordingly, depletion of NK cells impaired the elimination of B16 melanoma cells in the lungs of challenged young and aged mice, as determined by both quantitative RT–PCR expression of OVA (Fig.1B) and flow cytometric enumeration of CFSE+ cells (Fig.1C). Importantly, 1 h post-transfer, aged mice had a lung tumor burden comparable to that of anti-asialo GM1-treated mice and significantly higher than untreated young mice. Of note, there was a trend toward an increase in the number of NK cells in the lungs of challenged mice and this number was similar in both young and aged mice, which suggests NK cell homing to the lung in response to B16 melanoma challenge is not affected in aged mice (Fig.1D).

### Aged NK cells exhibit maturation defects and have a pronounced impairment in degranulation but not IFN-γ production after poly (I:C) priming or IL-2 activation

Consistent with previous findings, the proportion of mature NK cells (defined as CD3− NKp46+ CD27^low^ CD11b^hi^) was decreased in all lymphoid organs of aged mice (spleen, BM, LNs, lungs, liver and blood) (Fig. S1A and B and data not shown) (Nogusa *et al*., 2008, 2012; Fang *et al*., 2010; Beli *et al*., 2011, 2014; Chiu *et al*., 2013). We also confirmed these results using the expression of both CD43 and KLRG1, markers that are commonly used to identify terminally mature NK cells (Fig. S2). The frequency of immature stage D (CD11b^−^ CD43^−^) was augmented, while KLRG1+ NK cells were decreased in the spleen of aged mice (52.1%±1.7% in young vs. 27.4%±6.0% in aged, *P* = 0.007) and BM (14.5%±0.9% in young vs. 5.1%± 0.6% in aged, *P* = 0.0002).

**Figure 2:**
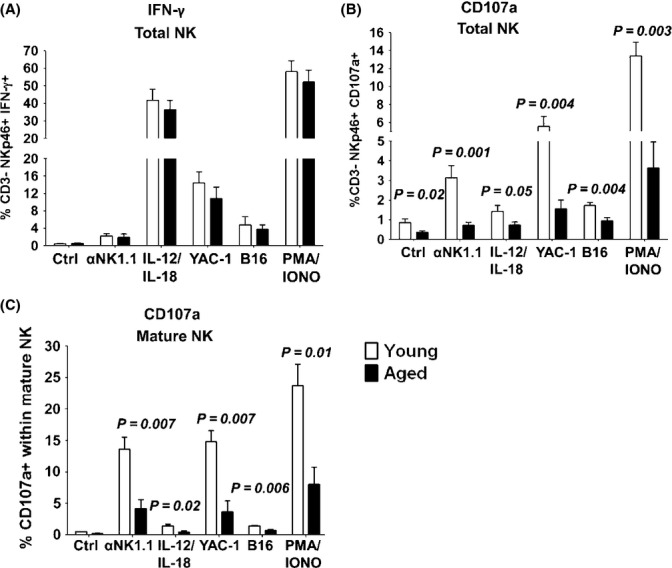
Aged NK cells have an impaired capacity to degranulate particularly in the mature NK cell subset. Aged and young mice were treated intraperitoneally with 100 μg of poly (I: C), and after 24 h, 10^6^ splenocytes were stimulated with various stimuli such as plate-bound anti-NK1.1 (10 μg/mL), IL-12 (1 ng/mL)/IL-18 (20 ng/mL), 2 × 10^5^ B16 melanoma cells, 2 × 10^5^ YAC-1 cells and PMA (50 ng/mL)/Ionomycin (750 ng/mL) for 5 h before analysis of CD107a surface expression and intracellular IFN-γ within the CD3- NKp46+ population. (A) The proportion of total splenic NK cells is IFN-γ+. CD107a degranulation was assessed in total splenic NK cells (CD3- NKp46+), (B) and mature NK cells, (C), of aged and young mice. Bar graphs show mean± SE of 8 aged and 7 young mice in two experiments. The *P* values represent the difference between aged and young mice (unpaired *t*-test).

It is well known that the mature NK cell subset has a higher expression of genes involved in NK cell effector functions and a higher overall functionality (Kim *et al*., 2002; Fang *et al*., 2008; Chiossone *et al*., 2009). Thus, in addition to defects at a population level (i.e., mature vs immature), it was also possible that aged NK cells are impaired in their cytotoxic capacity. To test this, we primed NK cell activity using poly (I:C) *in vivo* and evaluated the potential of aged NK cells to degranulate and produce IFN-γ in response to various NK cell stimuli *in vitro*. Aged NK cells exhibited a comparable capacity to produce IFN-γ as young NK cells (Fig.2A). In contrast, a significant decrease in surface expression of the degranulation marker CD107a (LAMP-1), but not in its intracellular levels (data not shown), suggests that aged NK cells have an impaired capacity to degranulate following their *in vivo* priming (Fig.2B). Similar findings were observed when NK cells were activated *in vitro* by IL-2 (data not shown). Importantly, this defect was particularly pronounced in the transitional and mature, but not immature subsets of NK cells that expressed very low levels of CD107a as expected (Fig.2C and data not shown) (Kim *et al*., 2002; Chiossone *et al*., 2009). Therefore, both the reduced proportion of mature NK cells and functional defects in mature NK cells may contribute to the impaired *in vivo* cytotoxicity of NK cells in aging.

### The aged nonhematopoietic environment limits NK cell maturation

**Figure 3:**
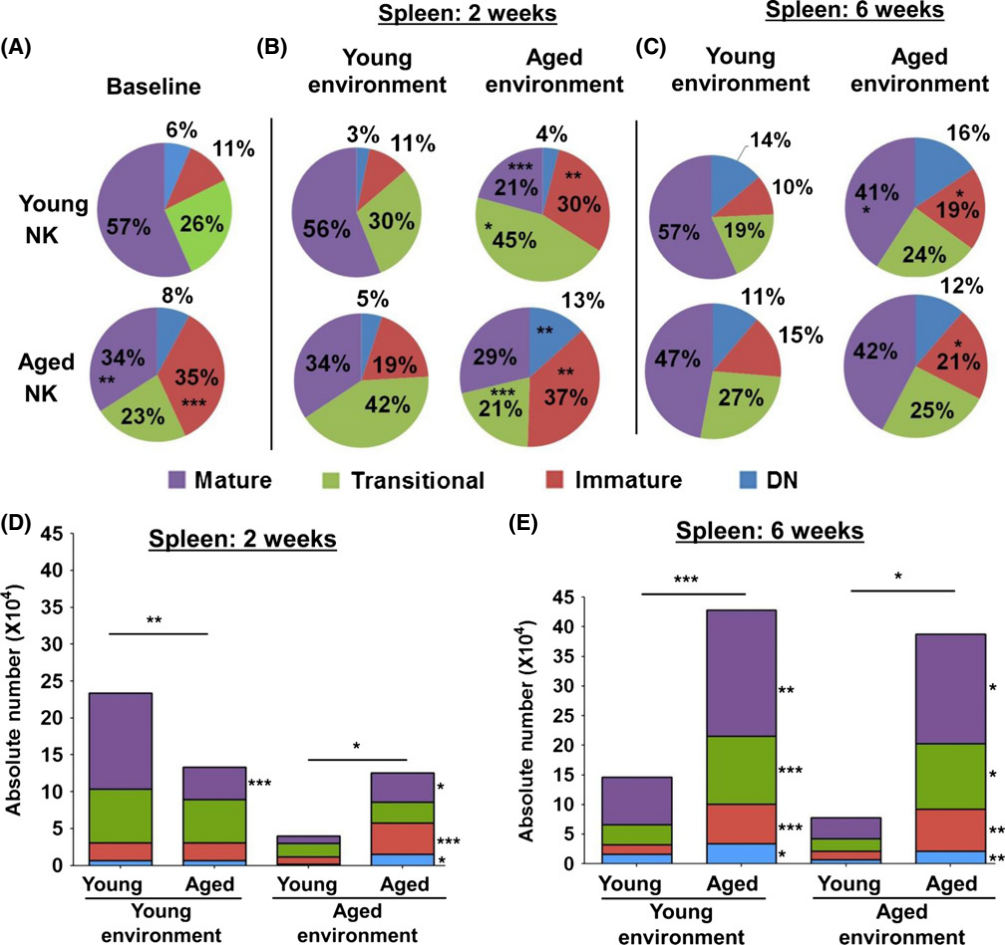
The aged environment plays an important role in limiting NK cell maturation *in vivo*. Analysis of splenic NK cell maturation at baseline, (A) or 2 weeks, (B) and 6 weeks, (C)*,* postchimerism in a young or aged environment. (D) and (E)*,* Absolute numbers of NK cells and their maturation subsets after developing in a young or aged environment at 2 weeks or 6 weeks postchimerism. Numbers in pie charts represent the mean of the proportion of each subset. Data shown are representative of at least two independent experiments with *n = *6 mice per group. The *P* values for week 2 and week 6 of the mixed BM chimera represent the difference between NK cells maturing in a young vs. aged environment, while the *P* values at baseline conditions represent the differences between NK cells from young and aged mice (unpaired *t*-tests), *P < 0.05, **P < 0.009, and ***P < 0.0005.

To determine the underlying mechanism(s) leading to the reduced maturation of aged NK cells, we asked whether NK cell intrinsic or extrinsic defects were responsible. We constructed mixed BM chimeras in which T-cell-depleted BM cells from aged (CD45.2+) and young (CD45.1+) donors were mixed at a 1:1 ratio and adoptively transferred into young (CD45.1+) or aged (CD45.2+) recipients (Fig. S3A) and compared the development of NK cells from both sources in the same environment. Intriguingly, despite aged and young NK cells being provided in the BM inoculum at a 1:1 ratio (Fig. S3B), NK cell chimerism in the BM and periphery was not proportionally established in both environments at weeks 2 and 6 postchimerism as assessed by the absolute number (Fig3D and E). At week 2, there was a dominance of young NK cells in the periphery of young recipients (Fig.3D). In contrast, in aged recipients, chimerism was established in favor of NK cells from an aged origin (Fig3D). Surprisingly, at week 6, aged NK cells were dominant in both young and aged recipients (Fig.3E). However, this dominance in chimerism was also observed for the whole population of cells derived from an aged origin (CD45.2+), suggesting that the dominance in chimerism was not NK cell specific (Fig. S3C and D).

The maturation status of NK cells from both origins was evaluated 2 and 6 weeks postchimerism in both a young and aged environment based on their respective congenic markers. NK cells from an aged origin had an augmented maturation that was similar to that of young donor NK cells (baseline) when they developed in a young environment at 2 weeks postchimerism in the spleen and BM (Fig.3B and C and data not shown). In contrast, NK cells from a young origin developing in an aged environment acquired a maturation phenotype reminiscent of that of aged donor NK cells (baseline) in both the spleen and BM (Fig.3B and C and data not shown) 2 weeks postchimerism. While young and aged NK cells developing in an aged environment had an impaired maturation compared to those developing in a young environment at 6 weeks postchimerism, these differences were more subtle compared to those observed at 2 weeks. Interestingly, the presence of young BM cells in the BM inoculum did not augment aged NK cell maturation in sublethally irradiated aged recipients and nor did the presence of aged BM cells impair the maturation of young NK cells in sublethally irradiated young recipients. These novel and important findings suggest that the nonhematopoietic environment in aged recipients plays a role in contributing to the impaired maturation of NK cells.

### Eomes and T-bet are significantly reduced in aged BM NK cells and are regulated by the environment in which NK cells develop

**Figure 4:**
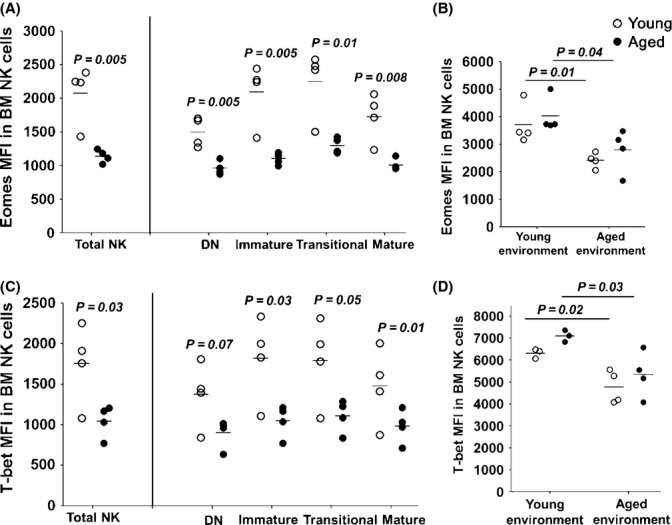
Eomes and T-bet, which regulate key checkpoints in NK cell functional maturation, are significantly reduced in aged BM NK cells. (A) and (C), Eomes and T-bet intracellular expression in young and aged NK cells in the bone marrow. The mean fluorescence intensity (MFI) of Eomes, (A) and T-bet, (C), in each of the young and aged NK cell subsets in the BM. (B) and (D), The MFI of T-bet and Eomes in whole young or aged BM NK cells that developed in either a young or aged host. Data are representative from at least two independent experiments with each point representing one individual mouse. Mean MFI is shown as horizontal bars. The *P* values represent the difference between aged and young NK cells (unpaired *t*-test).

Previous studies have shown that the transcription factors, T-bet, Eomes, GATA-3, and Blimp-1, play a critical role in directing and modulating NK cell functional maturation (Samson *et al*., 2003; Townsend *et al*., 2004; Kallies *et al*., 2011; Gordon *et al*., 2012). To determine whether the defects in NK cell maturation and function were associated with a reduction in expression of these key transcription factors, we evaluated their intracellular expression by flow cytometry. Expression of the transcription factors, Eomes and T-bet (Fig.4A and C) but not GATA-3 or Blimp-1 (Table S1), was significantly decreased in aged BM NK cells and their subsets. Importantly, in our mixed BM chimera studies, the development of aged NK cells in a young environment restored both Eomes and T-bet levels (Fig.4B and D). Thus, NK cells from an aged origin maturing in a young environment had an expression of both T-bet and Eomes, comparable to those of NK cells from a young origin, but significantly higher than that in aged NK cells maturing in an aged environment. In contrast, development of young NK cells in an aged environment led to the downregulation of both Eomes and T-bet (Fig.4B and D).

T-bet and Eomes have been shown to control NK cell maturation and NK cell cytotoxicity by regulating the expression of perforin and granzyme B (GZB) (Townsend *et al*., 2004). Consistent with decreased T-bet and Eomes, we found that the proportion of GZB+ NK cells was significantly reduced in the spleen of aged mice (28%± 7% vs. 60%± 12% in young mice, *P* = 0.004).

One of the major factors implicated in regulating T-bet and Eomes expression and NK cell maturation is IL-15 as well as the interaction between NK cells and stromal cells (Townsend *et al*., 2004; Caraux *et al*., 2006; Roth *et al*., 2007). Similar levels of IL-15mRNA were found in whole BM cells (4.3 ± 0.4 in young vs. 4.07 ± 0.4 in aged, *P* = 0.6) and splenocytes (5.49 ± 0.78 in young vs. 8.24 ± 1.72 in aged, *P* = 0.22) of aged and young mice. Although the MFI of CD122 was significantly, but modestly, decreased in aged NK cells (mean 22% decrease in aged NK cells, *P* = 0.001), the *in vitro* response to IL-15 was similar for aged and young NK cells (Fig. S4A). Additionally, the surface expression (MFI) of the IL-15Rα which is essential for the transpresentation of IL-15 to NK cells was similar between aged and young mice when assessed by flow cytometry in whole splenocytes, BM cells as well as splenic macrophages (F480+ CD11b+), dendritic cells (DCs= Lin- CD11c+ MHC-II+), and the different DC subsets (CD8α-CD11b-, CD8α+ CD11b- and CD8α- CD11b+) (data not shown). Importantly, administering a large quantity of IL-15/IL-15Rα complex to aged mice did not augment NK cell maturation in the BM (Fig. S4B), although the IL-15/IL-15Rα complex promoted the expansion of aged NK cells similar to that of young NK cells (data not shown). Together, the data suggest that deficiencies in IL-15 production or the impaired capacity for IL-15 transpresentation or IL-15 signaling in aging are unlikely to be the mechanisms underlying the impaired maturation of NK cells in aging.

### Aged NK cells developing in a young environment have an enhanced functional capacity comparable to young NK cells

**Figure 5:**
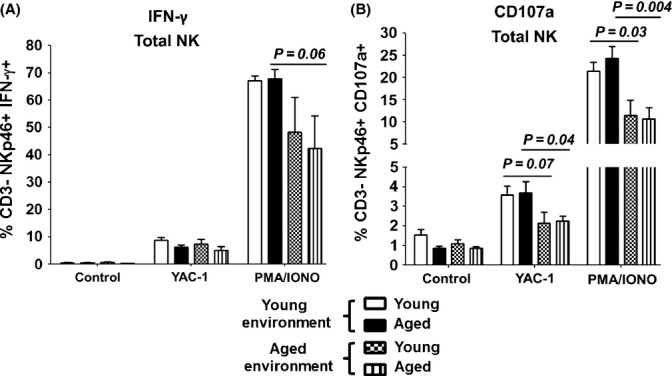
The functional capacity of aged NK cells is augmented when they mature in a young environment. Six weeks postchimerism, splenocytes were isolated from recipients of mixed BM chimeras and cultured in the presence or absence of 2 × 10^5^ YAC-1 cells or PMA (50 ng/mL)/Ionomycin (750 ng/mL) for 5 h in addition to IL-2 (500 U/mL). Intracellular IFN-γ, (A) and CD107a degranulation, (B), were assessed 5 h poststimulation in aged and young total NK cells*,* based on expression of their respective congenic markers. Bar graphs show mean± SE of 6 aged and 6 young mice. The *P* values represent the difference between aged and young NK cells (unpaired t-test).

It had never been shown whether the nonhematopoietic environment in which NK cells mature dictates their functionality. Thus, we assessed the capacity of aged and young splenic NK cells developing in both aged and young hosts to degranulate and produce IFN-γ in response to robust PMA/Ionomycin and YAC-1 stimulation. Interestingly, aged NK cells developing in young hosts had improved their capacity to degranulate and produce IFN-γ, reaching levels similar to that of young NK cells developing in the same environment (Fig.5). Furthermore, this enhanced functional capacity was particularly apparent in the mature NK cell subset from an aged origin (data not shown). The aged environment had the reverse effect, in that NK cells from a young origin developing in an aged environment had an attenuated capacity to degranulate and produce IFN-γ similar to their aged counterparts, but substantially less than those developing in a young environment (Fig.5).

## Discussion

Aging influences many aspects of both innate and adaptive immunity. Here, our studies provide a novel understanding of how aging influences NK cell maturation and function by revealing that the aged environment and, in particular, the nonhematopoietic compartment plays a critical role in impairing the maturation and function of NK cells in aging. This offers new insight that may provide leads on therapeutic interventions to reverse the NK cell phenotype in aging.

As demonstrated here and elsewhere (Nogusa *et al*., 2008; Fang *et al*., 2010; Beli *et al*., 2011, 2014), aging significantly impairs the maturation and the cytotoxic capacity of human and murine NK cells *in vitro*. We extended our studies to show that the cytotoxicity of murine NK cells is impaired *in vivo* against allogeneic and B16 melanoma tumor cells. Most studies using this latter model evaluate lung tumor burden (nodules) at least 2 weeks postchallenge in young mice, but this model is not useful to assess NK cell cytotoxicity in aged mice because in these mice, the number of developing metastatic lung colonies is much lower than in young mice, a phenomenon attributed to impaired vascularity and tumor angiogenesis (Pettan-Brewer *et al*., 2012). We therefore used the model described by Grundy and colleagues in which B16 tumor cells are rapidly destroyed by NK cells in the lung (as early as 50 min postinjection) with NK cell numbers peaking 20 min postchallenge (Grundy *et al*., 2007). In the context of ectromelia virus infection (ECTV), aged NK cells have an impaired capacity to migrate to draining lymph nodes (Fang *et al*., 2010). It was thus possible that a similar phenomenon could be contributing to the reduced elimination of B16 tumor cells in the lung of aged mice. However, we found similar NK cell absolute numbers in the lung tissue of young and aged mice post-B16 challenge, suggesting that the reduced elimination of B16 tumor cells in aged mice was not due to defects in NK cell homing to the lung, but suggested a functional deficit of NK cells in aging. Consistent with the fact that the mature NK cell subset displays the full repertoire of Ly49 receptors and has the highest cytotoxic potential, the reduced frequency of this subset in the peripheral lymphoid organs of aged mice is a major contributor to the overall reduced effector functions of aged NK cells *in vitro* and *in vivo*.

At a subset level, the impaired capacity of aged NK cells to degranulate following *in vivo* priming with poly (I:C) had never been shown. We observed impaired degranulation mostly in the mature NK cell subset of aged mice, highlighting that not only does the reduced frequency of the mature NK cell subset contribute to the overall reduction in aged NK cell functionality, but that mature NK cells in aging are also hyporesponsive. Accordingly, reduced cytotoxicity against multiple targets and reduced GZB expression was found in aged NK cells. Mechanistically, we found that the reduced protein expression of the transcription factors, T-bet and Eomes which have been shown to regulate perforin and GZB, may play an important role in reducing the cytotoxic potential of aged NK cells. Indeed, a reduced proportion of aged NK cells are GZB+ and T-bet^−/−^ mice are highly susceptible to B16 melanoma (Werneck *et al*., 2008). Thus, determining the factors influencing T-bet and Eomes expression may provide promising therapeutic interventions to augment the function of NK cells in aging.

IL-15 and the interaction between NK cells and stromal cells are key factors regulating T-bet and Eomes expression in NK cells (Townsend *et al*., 2004; Caraux *et al*., 2006; Roth *et al*., 2007). IL-15 is the essential NK cell fuel that promotes both the survival and functional maturation of NK cells when it is transpresented by DCs, macrophages, and stromal cells (Cooper *et al*., 2002; Huntington *et al*., 2007; Lee *et al*., 2011). In mice lacking IL-15 or its receptors, the numbers of NK cell progenitors are relatively normal, but the maturation and cytotoxic capacity of peripheral NK cells are severely reduced compared with control animals (Vosshenrich *et al*., 2005). Furthermore, the interaction of NK cells with CXCL-12 abundant reticular BM stromal cells (CAR cells), which express high levels of IL-15, has also been shown to play an important role in promoting the maturation and function of NK cells (Noda *et al*., 2011; Bernardini *et al*., 2013). Thus, defining whether a reduced expression of IL-15 was evident in aging was imperative. If IL-15 was the limiting factor in aging, then administering IL-15 would compensate for this deficiency and augment NK cell maturation in aging. However, a lack of IL-15 does not appear to be responsible for the impaired maturation of NK cells in aging as administering the IL-15/IL-15Rα complex *in vivo* did not augment BM NK cell maturation in aged mice. These data are in agreement with the fact that the number of IL-15+ as well as IL-15 levels increased in splenic stromal cells from middle-aged mice (10 month old) compared to young mice (Cui *et al*., 2014). Furthermore, we show that IL-15 signaling or the impaired capacity for optimal IL-15 transpresentation through the IL-15Rα do not appear to be impaired in aging.

Interestingly, our findings highlight that the environment in which NK cells mature governs the expression profile of both T-bet and Eomes. Notably, the presence of young BM cells in the mixed BM chimera inoculum did not contribute to the augmented maturation and function of aged NK cells in sublethally irradiated aged hosts 2 weeks postchimerism. Similarly, the presence of aged BM cells in the BM inoculum did not impair the young NK cell maturation and function in sublethally irradiated young hosts 2 weeks postchimerism. While there were trends toward a similar pattern at 6 weeks postchimerism, the differences at 6 weeks were more subtle than those at 2 weeks. Therefore, while the aged nonhematopoietic environment may impair NK cell maturation, other factors including defects in the hematopoietic environment as well as intrinsic defects in hematopoietic stem cells (HSCs) and NK cell precursors may also contribute to the impaired NK cell maturation and function in aging. Together, these results advance our understanding of how aging influences NK cells by revealing the contribution of the aged nonhematopoietic environment on the impaired maturation and function of NK cells. These data are in agreement with those from Chiu *et al*. (2013). However, we significantly extended these analyses and showed that the expression of T-bet and Eomes as well as the functional capacity of aged NK cells is also controlled by the nonhematopoietic environment. Our data, thus, suggest that therapeutic interventions geared at boosting NK cell cytotoxicity in aging will need to manipulate the nonhematopoietic environment in which NK cells develop. Indeed, our data highlight that aged NK cells are intrinsically capable of being functionally competent provided they undergo maturation in a ‘young’ environment. The use of stem cell transplantation as a therapeutic intervention to reverse immune dysfunction in aging has received a growing amount of interest (Zhao *et al*., 2013). Thus, integrating stem cell therapy with the transfer of young nonhematopoietic cells or a way to improve the aged environment may have considerable promise in reversing the phenotype of NK cells in aging.

An intriguing finding in our analysis was the observation that in mixed BM chimeras, NK cells from an aged origin dominated the NK cell compartment in the periphery (spleen, pLNs, and lungs) despite the BM inoculum having a 1:1 ratio of aged and young NK cells. However, this phenomenon was not specific to NK cells as bulk CD45.2+ aged cells dominated the hematopoietic compartment in both young and aged recipients at week 6 postchimerism. While the mechanism to account for this phenomenon is yet to be defined, one explanation for these results could be an enhanced survival capacity of aged NK cells *in vivo* which allows them to dominate in cellular competition. Indeed, preliminary results show that aged splenic NK cells have a higher (twofold) protein expression of the pro-survival molecule, Mcl-1, but the expression of Bcl-2 and Bim is similar between young and aged splenic NK cells (data not shown). Similarly, aged naïve T cells have been shown to have an enhanced survival compared to young cells when transferred in the same host (Tsukamoto *et al*., 2009). This suggests that cells from aged donors may have an intrinsically enhanced capacity to survive in the same environment compared to cells from young donors. An alternative explanation could be enhanced aged NK cell proliferation or skewed migration between peripheral lymphoid organs. However, NK cell proliferation as measured by the expression of the nuclear proliferation antigen, Ki-67 was similar between aged and young NK cells in our mixed BM chimera experiments (data not shown). Additionally, the dominance of NK cells from an aged origin was present not only in the spleen, but also pLNs, BM, and the lung suggesting that preferential migration was unlikely to explain this phenomenon.

In conclusion, in this study, we demonstrate that the aged nonhematopoietic environment is an important contributor to the impaired maturation and function of NK cells in aging. These findings open up the possibility of therapeutic interventions geared at boosting the functional competence of NK cells against tumors and viral infections, both of which occur at a high incidence in the elderly; therefore, identifying the nonhematopoietic environmental defects that exist in aging is of high significance to human health.

## Experimental procedures

### Mice

C57BL/6 female mice were purchased from either Taconic Farms (Germantown, NY, USA), The Jackson Laboratory (Bar Harbor, ME, USA), or from the National Institutes of Aging (NIA) colony at Taconic Farms. Young mice were considered to be 2–3 months of age, and aged mice were considered to be ≥ 16 months of age. All mice were acclimatized for at least 1 week before conducting any studies. Mice were housed under specific pathogen-free conditions in the Cincinnati Children's Hospital Medical Center (CCHMC) vivarium. Congenic CD45.1 C57BL/6 mice were obtained from Jackson Laboratories. All animal protocols were reviewed and approved by CCHMC Institutional Animal Care and Use Committee.

### Cell preparations

#### Bone marrow cell preparation

The hind legs were dissected and carefully removed from the surrounding muscle tissue. The femurs and tibia were then crushed using a mortar and pestle in 10 mL of phosphate buffered solution (PBS, Mediatech, Manassas, VA, USA) containing 2% FBS (Invitrogen, Carlsbad, CA, USA). The obtained solution containing the bone marrow was filtered on a 70-μm cell strainer and washed twice before red blood cell lysis with Ammonium-Chloride-Potassium (ACK) (prepared in house).

#### Spleen and peripheral lymph node cell preparation

Spleens and peripheral lymph node (pLNs) (inguinal, axillary, brachial, and cervical) were homogenized with a 3-mL syringe plunger through a 100-μm cell strainer using buffered saline solution (BSS) (prepared in house), and red blood cells were lysed with ACK lysis buffer (prepared in house).

#### Lung cell preparation

Lungs were isolated and digested in an enzyme mixture of 6 mL of RPMI 1640 containing Liberase CI (0.5 mg/mL) (Roche Diagnostics, Indianapolis, IN, USA) and DNase I (0.5 mg/mL) (Sigma-Aldrich, St. Louis, MO, USA) for 45 min at 37 °C. The digested lung tissue was then homogenized with a 3-mL syringe plunger through a 70-μm cell strainer, and red blood cells were lysed with ACK lysis buffer (prepared in house).

#### Liver cell preparation

Single liver cell suspensions were prepared by first homogenizing the whole tissue in RPMI 1640 using a gentle MACS dissociator (Miltenyi Biotech, Auburn, CA, USA). The resulting homogenate was then centrifuged at 931 g, and the cell pellet was mixed with 33% Percoll (Sigma-Aldrich) in RPMI 1640 solution (Invitrogen). The cell suspension was then centrifuged at 931 g for 20 min at room temperature. The cell pellet was removed and washed, and red blood cells were lysed with ACK lysis buffer (prepared in house). Single cell suspensions from all tissues analyzed were then washed with RPMI 1640 (Invitrogen) containing 10% FBS, and viable cells were counted via trypan blue (MP Biomedicals, Solon, OH, USA) exclusion.

### *In vivo* NK cell cytotoxicity assay against allogeneic and B16 melanoma targets

Aged and young mice were injected intravenously (i.v) with a 1:1 mixture of a low carboxyfluoresceinsuccinimidyl ester (CFSE) (Life technologies, Grand Island, NY, USA) syngeneic C57BL/6 splenocytes (2 μm CFSE) reference population and a medium CFSE allogeneic Balb/c splenocytes (10 μm CFSE) target cell population (10^7^ total cells). Eighteen hours after injection of the CFSE-labeled cell mixture, a blood sample was taken from each recipient mouse and the presence/absence of the reference and target cell populations was determined by flow cytometry.

NK cell depletion *in vivo* was performed by injecting mice intraperitoneally with 20 μL of anti-asialo GM1 antibody as recommended by the manufacturer (Wako Pure Chemical Industries, Richmond, VA, USA). Administration of anti-asialo GM1 was performed twice, on day 3 and 24 h before challenge of recipients with target cells. NK cell cytotoxicity was determined by normalizing the percentage of target cell killing in relation to NK cell-depleted recipients (>90% depletion in anti-asialo GM1-treated recipient mice). Assessment of B16 melanoma lung tumor burden was performed 1 h after i.v injection of 0.2 × 10^6^ (10 μm CFSE) labeled B16 melanoma cells expressing OVA using both flow cytometry (for detection of CFSE+ cells) and RT–PCR (for detection of OVA expression)(Grundy *et al*., 2007). The right lung was isolated and digested in a Liberase (Roche Diagnostics)/DNase (Sigma-Aldrich) enzyme mixture as described above.

### Antibodies and flow cytometry

10^6^ cells from single cell suspensions were suspended in 100 μL fluorescence-activated cell sorting buffer, and Fc receptor was blocked with anti-mouse CD16/32 (clone 93, Biolegend). The following mAbs (purchased from eBioscience, San Diego, CA, BD, San Jose, CA, USA; Biolegend, San Diego, CA, USA or Cell Signaling, Technology, Beverly, MA, USA) were used: anti-CD3 (500A2), anti-NKp46 (29A1.4), anti-CD27 (LG.3A10 or LG.7F9), anti-CD11b (M1/70), anti-T-bet (ebio4B10), anti-Eomes (DAN11MAG), anti-Blimp-1 (C14A4), anti-GATA-3 (TWAJ), anti-CD45.2 (104), anti-CD45.1 (A20), anti-KLRG1 (2F1), anti-CD43 (S7), anti-CD107 (1D4B), anti-Ly49G2 (4D11), anti-Ly49I (YL1-90), anti-IFN-γ (XMG1.2), anti-granzyme B (GB11), anti-F4/80 (BM8), anti-CD8α (53-6.7), anti-CD11c (N418), anti-MHC-II (M5/114.15.2), anti-CD19 (6D5), anti-IL-15Rα (DNT15Ra), and anti-CD122 (TM-β1). For detection of Blimp-1, secondary anti-rabbit antibodies were used (Jackson ImmunoResearch Laboratories, West Grove, PA, USA). Lineage (Lin) is defined using CD3, CD19, and NKp46. NK cells are defined as CD3- NKp46+ within live cells (Live-Dead fixable blue negative, Life technologies), and NK cell subsets are defined based on expression of CD27 and CD11b as previously described(Hayakawa & Smyth, 2006; Walzer *et al*., 2007; Chiossone *et al*., 2009). DN NK cells are CD27^low^ CD11b^low^, immature NK cells are CD27^hi^ CD11b^low^, transitional NK cells are CD27^hi^ CD11b^hi^, and mature NK cells are CD27^low^ CD11b^hi^. Flow cytometry data were collected using an LSRII or LSR Fortessa (BD) flow cytometers and analyzed by bd facs diva software.

### Construction of Mixed BM chimeras

C57BL/6 recipient mice were lethally irradiated first with 700 rad and then with 475 rad 3 h later. After 24 h, recipient mice were injected intravenously with 10^7^ donor bone marrow cells containing a 1:1 mixture of young (CD45.1) and aged (CD45.2) bone marrow donor cells that were depleted of CD3+ TCRβ+T cells using CD3ε microbead kit (Miltenyi) and biotinylated TCRβ (Biolegend) and used according to the manufacturer's instructions. Recipient mice were then fed doxycycline-containing chow until the time of sacrifice. Chimerism was evaluated at 2 and 6 weeks post-transfer.

### NK cell degranulation assay

Young and aged mice were treated intraperitoneally with 100 μg of polyI:C. Spleens were harvested 24 h later, and 10^6^ splenocytes were cultured in the presence of either plate-bound anti-NK1.1 (PK136, 10 μg/mL, Biolegend), IL-12(p70) (1 ng/mL, PeproTech, Rocky Hill, NJ)/IL-18 (20 ng/mL, MBL, Boston, MA, USA), or 0.2X10^6^ YAC-1/B16 melanoma cells and phorbol myristate acetate (PMA) (Sigma-Aldrich) (50 ng/mL)/Ionomycin (Sigma-Aldrich) (750 ng/mL) for 5 h in the presence of brefeldin (Sigma-Aldrich) (2.5 mg/mL) and monensin (eBioscience) (1x). Similar experiments were conducted using IL-2 (Miltenyi Biotech) (500 U/mL) in the presence of the various stimuli indicated above.

### IL-15 administration *in vivo*

IL-15/IL-15Rα (4.5 μg) (R&D Systems, Minneapolis, MN, USA) were mixed *in vitro*, and the equivalent of 750 ng IL-15 was injected intraperitoneally on days 0, 2, and 4 before tissue harvest on day 6.

### Quantitative gene expression

RNA was prepared using the RNeasy Plus Mini kit (Qiagen, Valencia, CA, USA) and converted into cDNA using Superscript III Reverse Transcriptase (Invitrogen). Quantitative gene expression analysis (quantitative PCR) was performed with Roche LightCycler 480 SYBRGreen 1 Master Mix (Roche Diagnostics) using the Roche LightCycler 480 II instrument (Roche Diagnostics). The primers utilized were obtained from Integrated DNA Technologies (IDT, Coralville, IA, USA) and are as follows:

(i) Ribosomal protein s14 (Rps14) which was used as a house keeping gene for analysis of OVA expression in lung tissues: 5′-TGACATCCTCAATCCGCCCAATCT-3′ and 5′-CATCACTGCCTTGCACATCAAACT-3′; (ii) OVA: 5′-GTGACTGAGCAAGAAAGCAAACCTG-3′ and 5′-TTGTCCCACTGGCAAATGGAAG-3′; (iii) L19 as a house keeping gene for IL-15 analysis: 5′-CCTGAAGGTCAAAGGGAATGTG-3′ and 5′-GCTTTCGTGCTTCCTTGGTCT-3′ and (iv) IL-15: 5′-AACTGCTTTCTCCTGGAATTG-3′ and 5′-ATGAACATTTGGACAATGCGT-3′.

### Statistical analyses

Statistical analyses were performed using unpaired *t-*tests or nonparametric tests when appropriate. These tests were performed using graphpad prism software (version 5.01).

## Supporting Information

**Fig. S1.** Aged mice have a profound enrichment in immature NK cells across all lymphoid organs with a significant accrual in the BM.

**Fig. S2.***A*, The proportions of CD11b versus CD43 from gated splenic NK cells in the spleen and BM of young and aged mice respectively.

**Fig. S3.***A,* Construction of mixed BM chimeras in which a 1:1 mixture of aged and young BM cells were transferred i.v into either young or aged congenic recipients. *B,* The fraction of NK cells derived from both young and aged origins in the BM inoculum at the time of transfer into irradiated young and aged recipients.

**Fig. S4.** The response of young and aged splenic NK cells to different doses of IL-15 *in vitro*.

**Table S1.** Mean MFI±SE of GATA-3 and Blimp-1 in whole splenic and BM NK cells.
