# Supplementary material for: The aged nonhematopoietic environment impairs natural killer cell maturation and function
Source: Aging Cell. 2015 Feb 9;14(2):191–9. doi: 10.1111/acel.12303 (PMC4364831; doi:10.1111/acel.12303)
Supplement: Supplementary file 1 [file acel0014-0191-sd1.pdf]

Supplementary Figure 1

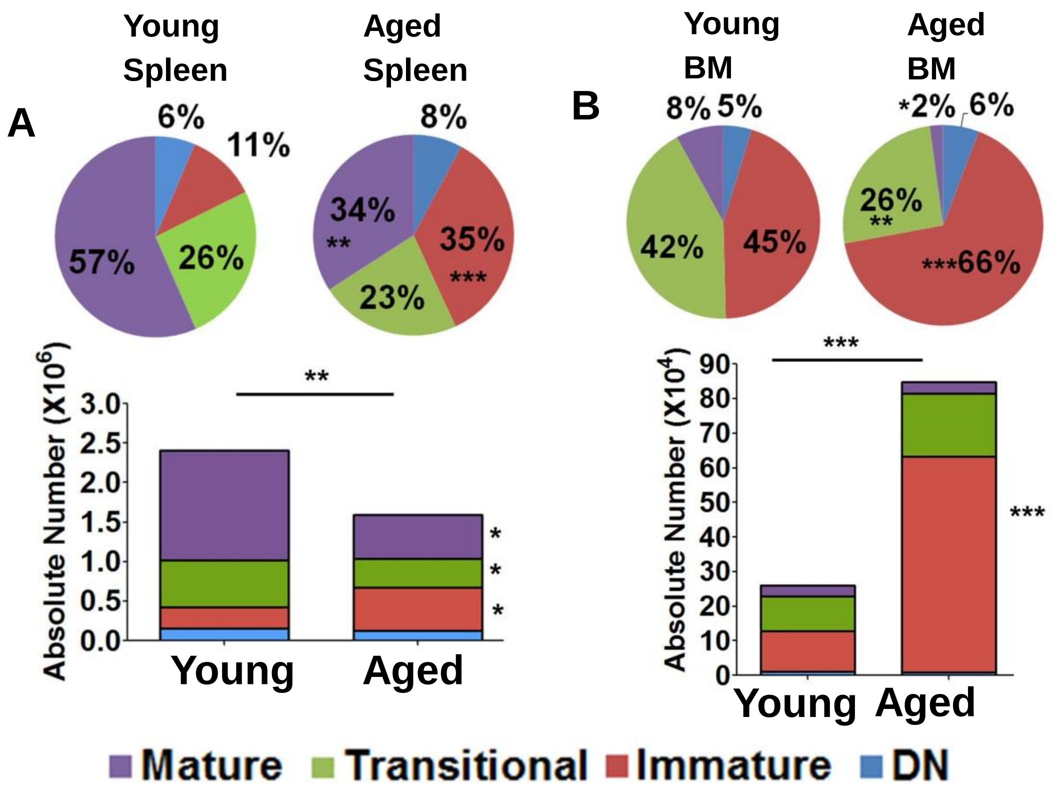

Fig. S1: Aged mice have a profound enrichment in immature NK cells across all lymphoid organs with a significant accrual in the BM. A, The proportion and absolute numbers of total NK cells across lymphoid organs in young and aged mice. The differences in proportion and absolute number of NK cell maturation subsets between young and aged mice in the spleen, B and the BM, C. All statistical analysis (unpaired t test) is performed in comparison to young NK cell subsets. Numbers in pie charts represent the mean of the proportion of each subset. Data are representative from two independent experiments with n=4 mice per group. Means are shown as horizontal lines with each point representing one individual mouse. The p values represent the difference between aged and young mice (unpaired t tests), *P<0.05, **P<0.009 and ***P<0.0005.
