# Supplementary material for: The aged nonhematopoietic environment impairs natural killer cell maturation and function
Source: Aging Cell. 2015 Feb 9;14(2):191–9. doi: 10.1111/acel.12303 (PMC4364831; doi:10.1111/acel.12303)
Supplement: Supplementary file 2 [file acel0014-0191-sd2.pdf]

Supplementary Figure 2

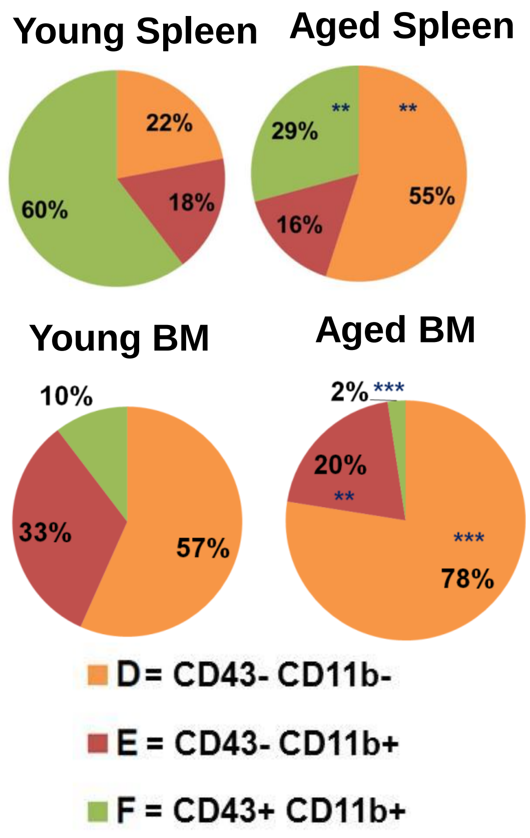

<u>Fig. S2: </u> *A*, The proportions of CD11b versus CD43 from gated splenic NK cells in the spleen and BM of young and aged mice respectively. Numbers in pie charts represent the mean of the proportion of each subset. The p values represent the difference between aged (N=4) and young (N=4) NK cells (unpaired t tests), *P<0.05, **P<0.009 and ***P<0.0005.
