# Supplementary material for: The aged nonhematopoietic environment impairs natural killer cell maturation and function
Source: Aging Cell. 2015 Feb 9;14(2):191–9. doi: 10.1111/acel.12303 (PMC4364831; doi:10.1111/acel.12303)
Supplement: Supplementary file 3 [file acel0014-0191-sd3.pdf]

Supplementary Figure 3

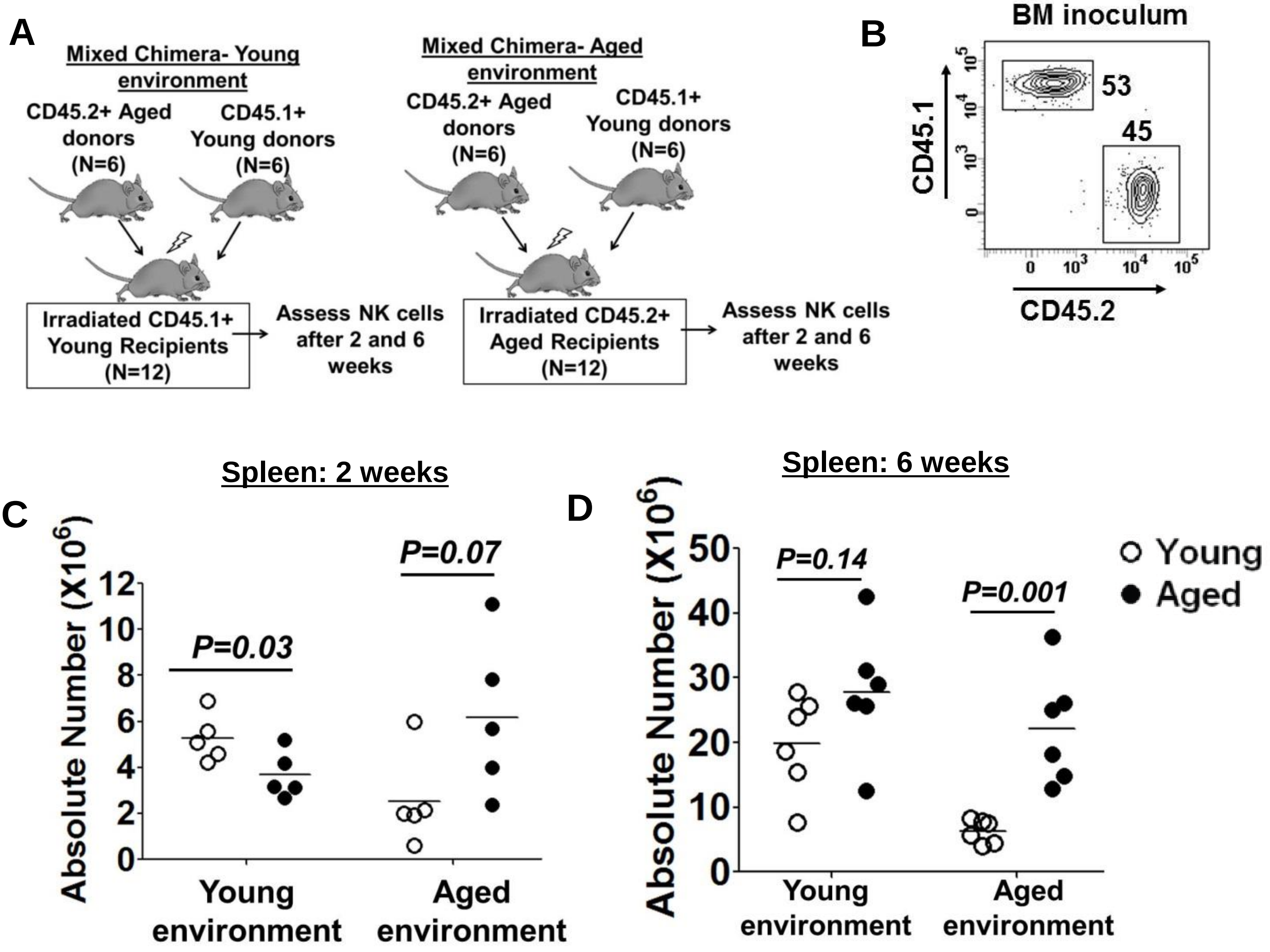

<u>**Fig. S**</u>3: *A*, Construction of mixed BM chimeras in which a 1:1 mixture of aged and young BM cells were transferred i.v into either young or aged congenic recipients. *B*, The fraction of NK cells derived from both young and aged origins in the BM inoculum at the time of transfer into irradiated young and aged recipients. This fraction was obtained by pooling BM cells from 6 young (CD45.1+) and 6 aged (CD45.2+) mice. The absolute numbers of total CD45.1+ (young) and CD45.2+ (aged) cells in the spleen 2 weeks (C) and 6 weeks (D) post chimerism.
