# Supplementary material for: The aged nonhematopoietic environment impairs natural killer cell maturation and function
Source: Aging Cell. 2015 Feb 9;14(2):191–9. doi: 10.1111/acel.12303 (PMC4364831; doi:10.1111/acel.12303)
Supplement: Supplementary file 4 [file acel0014-0191-sd4.pdf]

Supplementary Figure 4

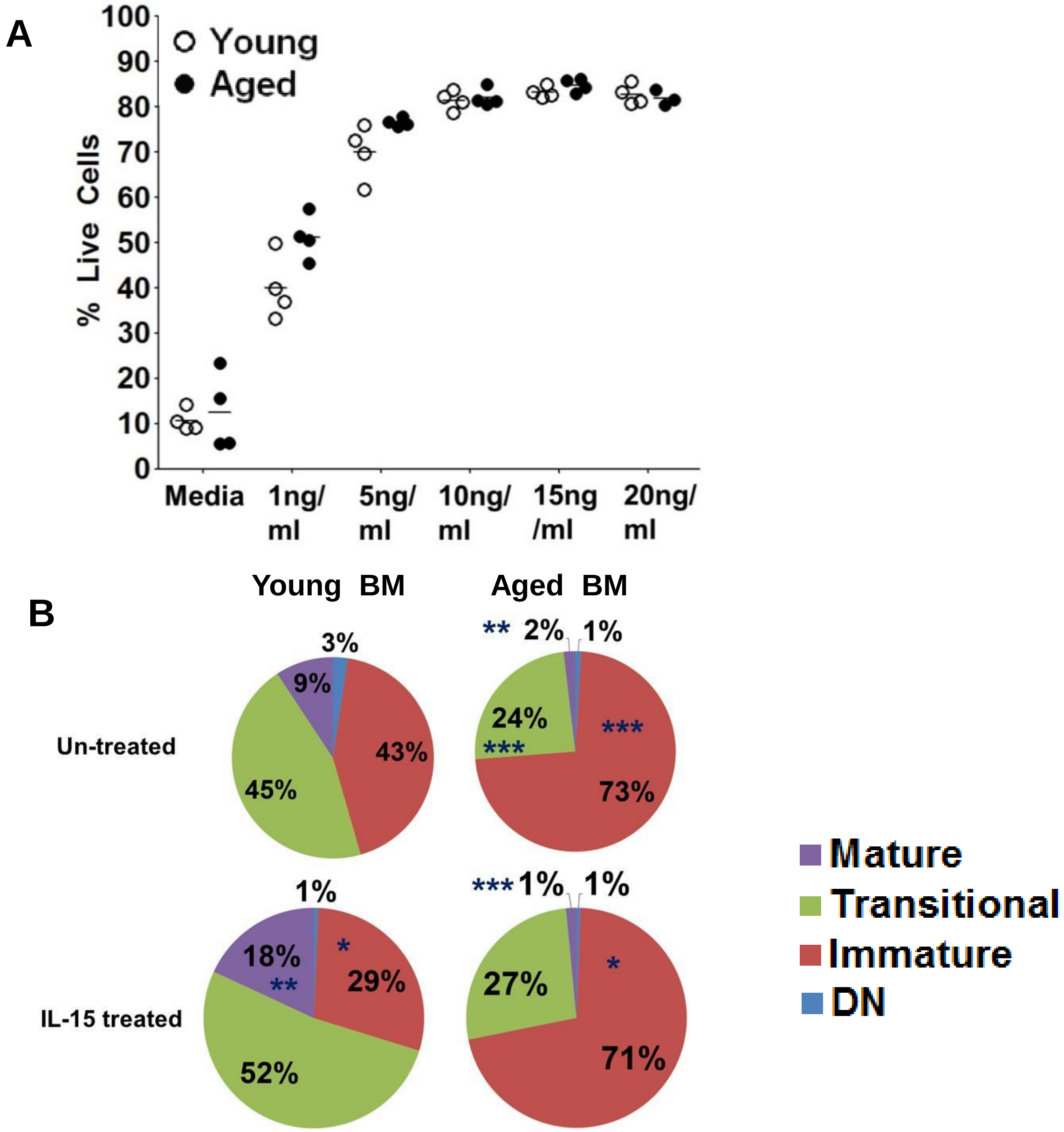

<u>Fig. S4</u>: The response of young and aged splenic NK cells to different doses of IL-15 *in vitro*. 10^6 splenocytes were cultured in the presence or absence (media) of different concentrations of recombinant mouse IL-15 for 24 hours before analysis of live cells (7AAD-) on gated CD3- NKp46+ cells. *B*, The maturation profile of aged and young BM NK cells with and without IL-15/IL-15Rα treatment. The p values represent the differences in relation to untreated young mice (unpaired t tests), *P<0.05, **P<0.009 and ***P<0.0005.
