# Supplementary material for: The aged nonhematopoietic environment impairs natural killer cell maturation and function
Source: Aging Cell. 2015 Feb 9;14(2):191–9. doi: 10.1111/acel.12303 (PMC4364831; doi:10.1111/acel.12303)
Supplement: Supplementary file 5 [file acel0014-0191-sd5.pdf]

Supplementary Table 1

	GATA-3		Blimp-1	
	Young	Aged	Young	Aged
Spleen	3077±139	3127±436	1122±20.28	1090±24.65
BM	884±59	802±52	881.8±54.71	720.8±50

Supplementary Table I: Mean MFI±SE of GATA-3 and Blimp-1 in whole splenic and BM NK cells. Data are representative from at least two independent experiments with 4 young and 4 aged mice. All p values representing the differences in GATA-3 and Blimp-1 expression between young and aged NK cells and their subsets are $p>0.07$.
